# Retrospective evaluation of labetalol as antihypertensive agent in dogs

**DOI:** 10.1186/s12917-020-02475-4

**Published:** 2020-07-24

**Authors:** Francesco Zublena, Chiara De Gennaro, Federico Corletto

**Affiliations:** Department of Veterinary Anaesthesia, Dick White Referrals, Six Mile Bottom, Station Farm, London Road, Six Mile Bottom, CB8 0UH Cambridgeshire, UK

**Keywords:** Labetalol, Arterial blood pressure, Dog, Craniotomy, Pheochromocytoma

## Abstract

**Background:**

To evaluate the effect on arterial blood pressure (ABP) of labetalol infusion as treatment for perioperative non nociceptive acute hypertension in dogs. The clinical records of dogs receiving intra or postoperative labetalol infusion were retrospectively reviewed. Invasive systolic (SAP), mean (MAP) and diastolic (DAP) arterial pressure and heart rate (HR) before labetalol infusion (T0) and 15, 30, 45 and 60 min (T1, T2, T3 and T4 respectively) after infusion were retrieved. The dose rate of labetalol infusion and use of concurrently administered drugs that could have potentially affected ABP and/or HR were also recorded. ANOVA for repeated measures and Dunnett’s multiple comparison test were used to determine the effect of labetalol on ABP and HR. Differences were considered significant when *p* < 0.05.

**Results:**

A total of 20 dogs met the inclusion criteria, and hypertension was documented after craniotomy (12/20), adrenalectomy (4/20) and other procedures (4/20). Five dogs received labetalol intraoperatively, 14 postoperatively, and 1 during the surgical procedure and recovery. Median infusion duration and rate were 463 (60-2120) minutes and 1.1 (0.2–3.4) mg/kg/h respectively. Median loading dose was 0.2 (0.2–0.4) mg/kg. Labetalol produced a significant decrease in SAP and DAP at all time points compared to T0 (*p* < 0.05), while the effect was not significant at T1 for MAP (*p* = 0.0519). Median maximum MAP decrease was 31 (20–90) mmHg. Heart rate did not increase significantly during treatment (*p* = 0.2454). Acepromazine given before or during labetalol treatment did not reduce significantly ABP (*p* = 0.735).

**Conclusions:**

Labetalol produced a reliable and titratable decrease in ABP with non significant increase in HR.

## Background

Labetalol hydrochloride (AH 5158) is a combined alpha- and beta-adrenergic blocking drug [1, 2]. It competitively antagonises the effect of both alpha (α_1_) and beta (β_1_ and β_2_) adrenergic stimulation by catecholamines and sympathetic nerves. This is a significant advantage when treating acute hypertension, as β- blockade alone leaves α-receptors catecholamine stimulation unopposed, with consequent compensatory increase of systemic vascular resistance. Similarly, α- blockade alone generally results in compensatory tachycardia. Labetalol decreases arterial blood pressure (ABP) and systemic vascular resistance, without significantly affecting cardiac output [3, 4]. The degree of α- blockade compared to the β- blocking effect has been investigated. Labetalol appears to be 4 times more potent in inhibiting β-adrenoceptors in anaesthetized dogs [2] and 3 to 7 times in humans [5]. A direct, non-adrenergic, vasodilator effect of labetalol has also been postulated in dogs [6].

Labetalol is a mixture of four stereoisomers present in equal proportion (RR, SR, SS, and RS). They block adrenergic receptors to varying degree, and the overall effect mostly depends on the combined action of RR and SR stereoisomers, which are potent antagonists of β- and α_1_ receptors respectively, while the activity of the other two stereoisomers is negligible [7, 8].

The effect of labetalol and other anti-hypertensive agents has been compared, demonstrating comparable efficacy and side effects profile to β-adrenergic blockers [9]. Labetalol is 2–10 times less potent than phentolamine in blocking α-adrenoceptors and 5–18 times less potent than propranolol in blocking β-adrenoceptors in dogs [2].

In human medicine labetalol is effective in reducing blood pressure in the short and long-term period, and the onset of effect during a continuous infusion is rapid and controlled [10, 11].

Systemic hypertension is a well-recognized complication during and after intracranial surgery in both humans and dogs, with an incidence of up to 57% reported in human medicine [12] and 52% in dogs [13]. Overzealous treatment of hypotension with vasopressors, pain induced sympathetic stimulation, fluid overload and a direct effect of brain manipulation have all been proposed as possible causes [14]. The common underlying mechanism appears to be sympathetic activation [15]. Hypertension after craniotomy can increase intracranial pressure, promote intracranial haemorrhage and cerebral oedema [16].

Adrenalectomy is also a surgical procedure carrying a significant risk of severe intra and postoperative hypertension in humans and dogs [17, 18]. Adrenalectomy is the treatment of choice for pheochromocytoma, a rare functional endocrine tumour of the adrenal medullary chromaffin cells, characterised by excessive release of catecholamines, resulting in haemodynamic instability and fluctuations of ABP and heart rate (HR) during anaesthesia [19].

To date, all published literature describing use of labetalol in dogs is limited to experimental models. Studies in anaesthetized dogs demonstrated the hypotensive effect of labetalol at the dose of 0.1 to 15 mg/kg intravenously [6, 20], and that a bolus of 100 mg followed by a constant rate infusion (CRI) of 30 mg/min produces a significative reduction in mean arterial pressure (MAP) without affecting intracranial pressure in experimental dogs weighing 27–33 kg [21]. Intravenous administration at doses of 0.1 to 3 mg/kg in anaesthetized dogs produced a dose-dependent reduction in blood pressure and myocardial contractility [2].

The aim of this manuscript is to report our experience using labetalol to control intra and postoperative hypertension in dogs in a clinical setting.

## Methods

Dogs receiving labetalol during hospitalisation between 2012 and 2017 were identified, searching the practice management system, and case records reviewed retrospectively, if the owner had not declined use of clinical data for research and educational purpose on the general consent form. Animals were included in this retrospective study if a hypertensive episode was documented either during surgery or in the immediate post-operative period, and was treated with labetalol (Labetalol Hydrochloride, Focus Pharmaceuticals, England). Dogs were excluded if medical records were incomplete or unclear, if blood pressure was not measured invasively, or if length of infusion was shorter than 60 min.

Data including signalment, procedure performed, HR, invasive ABP, body temperature, concomitant administration of drugs potentially affecting systemic or intracranial pressure (analgesics, acepromazine, dexmedetomidine, glycopyrrolate, steroids and mannitol) and postoperative analgesia were recorded on a spreadsheet. Precalibrated pressure transducers zeroed at the level of the heart (tip of the shoulder) were used to measure invasive blood pressure. They were sterilised between uses and regularly checked against a manometer.

The following information were collected for the period of labetalol administration, using a multiparameter monitor (PM-9000Vet, Mindray, UK; MostCare Up, Vygon, UK; and Beneview, Mindray, UK): systolic (SAP), MAP and diastolic (DAP) arterial pressure, HR and temperature at the time CRI of labetalol was started (defined as T0), the initial infusion rate and, if any, the loading dose of labetalol; SAP, MAP, DAP, HR and labetalol administration rate 15, 30, 45 and 60 min after infusion started (T1, T2, T3 and T4 respectively). Duration of labetalol infusion, the maximum and minimum infusion rate, the total amount administered, and the steps made to reduce it were also evaluated.

Due to the retrospective nature of the study, the decision of using labetalol to decrease ABP was completely at the anaesthetist’s discretion, both in terms of threshold for intervention and target value. The aim was to maintain MAP below 140 mmHg, as this was considered the value above which autoregulation is likely impaired. Intermittent positive pressure ventilation was used during anaesthesia to maintain normocarbia in all dogs. At the end of the procedure, once spontaneous breathing resumed, animals were transferred to the intensive care unit (ICU) and their vital parameters, including invasive ABP, were monitored continuously and treated as indicated by clinical condition. Postoperative analgesia was assessed every 2 h in all cases by a trained veterinary nurse or veterinary surgeon using the Short-Form of the Glasgow Composite Pain Scale, as per standard operating procedures in our practice [22]. Methadone was administered (0.2 mg/kg, IV or IM) either at fixed intervals of 4 h or as dictated by the assessment.

Monitoring of anaesthesia during surgery was performed by either an anaesthesia specialist or a resident, while data during the recovery phase were collected by a trained nurse, as part of the standard monitoring in our ICU. In all cases, use of labetalol was under the direct supervision of a senior anaesthetist.

All data were analysed using a commercially available statistical software program (Prism 8.0.1 for macOS, GraphPad Software, San Diego, California). Mixed effect ANOVA and a post hoc Dunnett’s multiple comparison test were used to analyse measured cardiovascular variables over time, before and during labetalol administration at different time points. The impact of acepromazine administration on blood pressure was assessed using Mann Whitney test. Results are expressed as mean (standard deviation) or median (range), according to the nature of the data and normality, tested using D’Agostino and Pearson Omnibus test. Differences were considered significant when *p* < 0.05.

## Results

**Fig. 1:**
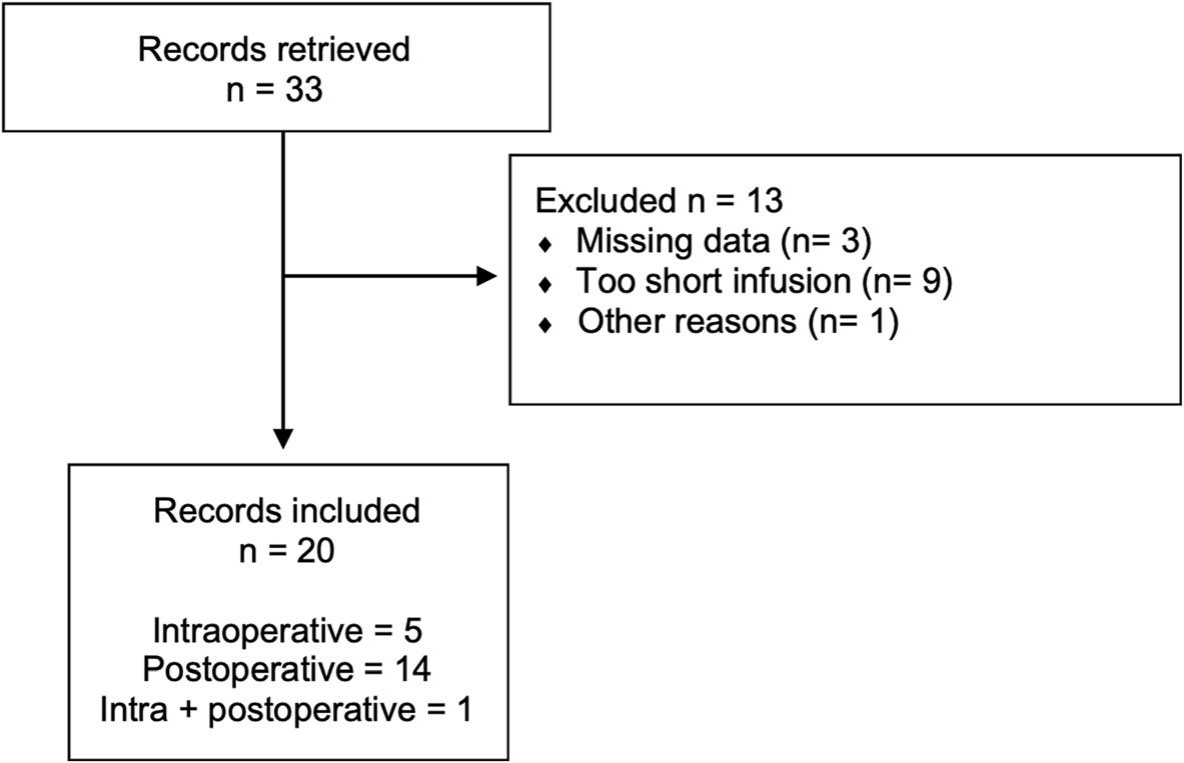
CONSORT diagram of dogs receiving labetalol infusion during or after surgery to treat non-nociceptive systemic hypertension

A total of 33 records of cases that received labetalol as part of the intra and/or post-operative management, were retrieved and analysed. Three cases were excluded due to incomplete records, one because of poor record keeping and nine because labetalol was infused for less than one hour. Twenty dogs were included in the study (Fig. 1). Breeds are presented in Table 1. Median weight was 27.6 (7.4–59.5) kg and median age 118 (25–180) months. Twelve (60%) patients underwent craniotomy for intracranial tumour removal or biopsy, 4 (20%) had adrenalectomy for excision of pheochromocytoma and the other 4 (20%) had an anaesthetic for gastric dilatation volvulus, liver lobectomy, trauma and cholecystectomy, respectively. An adrenal mass was found intraoperatively in the latter.

**Table 1:** Other drugs administered that may have affected blood pressure

Case	Breed	Weight (Kg)	Age (months)	Procedure	Analgesia	Other drugs that may affect blood pressure
1	Springer Spaniel cross	16.1	111	Craniotomy	DEX CRI	ACP, MAN, SEV
2	Flat-Coated Retriever	31	25	Craniotomy	DEX CRI	PRO CRI, MAN
3	Newfoundland	59.5	103	Craniotomy	DEX CRI, MET	MAN, ACP
4	Miniature Dachshund	7.4	132	Craniotomy	DEX CRI, MET, fentanyl, PAR	PRO CRI, MAN
5	Cross breed	13	144	Craniotomy	DEX CRI, MET, PAR	MAN
6	Border Terrier	12.4	132	Craniotomy	DEX CRI, MET, PAR	MAN, ACP, PHE
7	Boxer	24.2	74	Craniotomy	DEX CRI, MET	MAN, ACP, SEV
8	Cross breed	27.6	142	Adrenalectomy	Alfentanil CRI	SEV
9	Labrador	23	128	Adrenalectomy	MET	
10	Stabyhoun	26	106	Craniotomy	DEX CRI, MET, PAR	MAN, ACP, PHE
11	Cross breed	45.4	84	Craniotomy	MET	MAN, PHE
12	German Shepherd	32.5	147	Craniotomy	MET, PAR	MAN, ACP, PHE
13	Husky cross	33	125	Adrenalectomy	Epidural block (morphine + l-bupivacaine),	ACP, SEV, gelatins, phenoxybenzamine
14	Labrador	34.1	109	Adrenalectomy	Alfentanil and LID CRI	ISO, gelatins
15	Labrador	28.2	82	Craniotomy	DEX CRI, MET, PAR	MAN, ACP, PHE, furosemide
16	Border Terrier	8.2	180	Cholecystectomy (adrenal mass)	Epidural block (morphine + l-bupivacaine)	
17	Staffordshire Bull Terrier	21.4	144	Craniotomy	DEX CRI	MAN
18	Border Collie	23.8	150	Liver lobectomy	Epidural block (morphine + l-bupivacaine), MET, firocoxib	
19	Flat-coated Retriever	45	84	Gastric dilatation and volvulus	LID CRI, MET, PAR	
20	Bloodhound	50	31	Trauma management	MET CRI, meloxicam	

*DEX *Dexmedetomidine, *CRI *Constant rate infusion, *ACP *Acepromazine, *MAN *Mannitol, *SEV *Sevoflurane, *ISO *Isoflurane, *PRO *Propofol, *MET *Methadone, *PAR *Paracetamol, *PHE *Phenobarbital, *LID *Lidocaine

Pre-anaesthetic stabilization of the animal included administration of corticosteroids or other anti-inflammatory drugs, analgesics, antiemetics, antimicrobials, fluid therapy, anti-epileptic drugs and hyperosmolar agents, such as mannitol. One dog with pheochromocytoma received phenoxybenzamine treatment before surgery.

In case of intracranial surgery, according to the course of procedure and the intraoperative findings, mannitol (0.5 ± 0.01 mg/kg) was also administered (12/12 cases); 5 of them received phenobarbital (3.3 ± 1.7 mg/kg), and in 2 dogs dexamethasone (0.13 ± 0.01 mg/kg) was added to the other two drugs. In two cases transient intraoperative hypotension was treated with colloids (gelatins, 1.2 ml/kg bolus and 2 ml/kg/h). Intraoperative analgesia was achieved administering an infusion of dexmedetomidine (0.71 ± 0.27 mcg/kg/h), lidocaine (1.5 ± 0.7 mg/kg/h), an intravenous opioid (fentanyl 3.4 mcg/kg, alfentanil 40.2 ± 16.5 mcg/kg/h, and methadone 0.19 ± 0.05 mg/kg or 0.1 mg/kg/h), or an epidural block in some cases of abdominal surgery (L-bupivacaine and morphine, 0.8 ± 0.3 and 0.13 ± 0.05 mg/kg).

Average labetalol infusion rate was 1.1 (0.2–3.4) mg/kg/h and in 9 cases (45%) a loading dose of 0.2 (0.2–0.4) mg/kg IV was administered. Arterial blood pressure was continuously monitored during labetalol administration, and the dose of labetalol CRI was reduced by sequential steps in case of response, while it was increased if a greater decrease of blood pressure was deemed necessary.

Five (25%) animals received labetalol only intraoperatively, 14 (70%) postoperatively, and 1 (5%) received the infusion during the surgical procedure and recovery. Median infusion duration was 463 (60-2120) minutes.

The animals in which labetalol was infused intraoperatively were anaesthetized with isoflurane (1/6, mean end-tidal concentration 1.2%), sevoflurane (3/6, 2.2 ± 0.24%), or propofol (2/6, 16.9 ± 1.1 mg/kg/h).

**Fig. 2:**
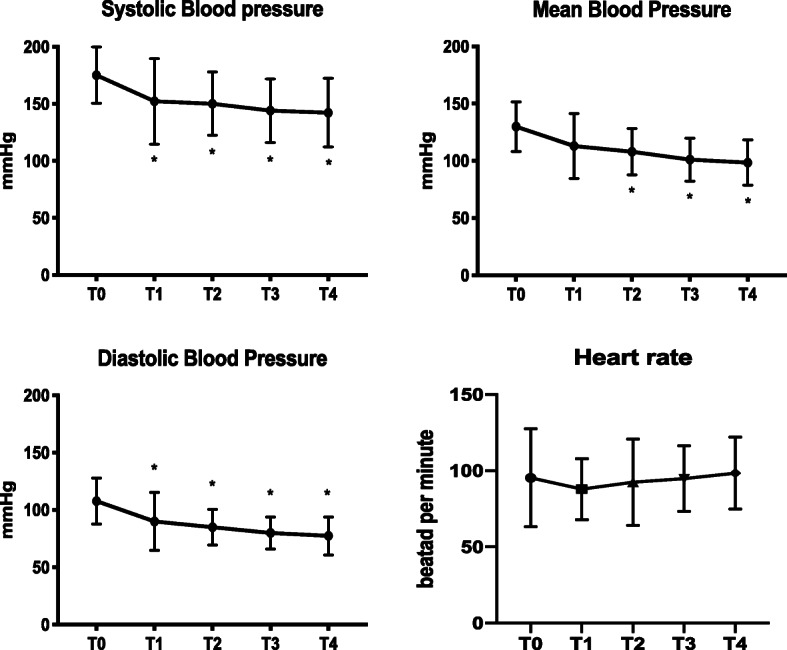
Change in cardiovascular parameters at the beginning of labetalol infusion (T0) and after 15 (T1), 30 (T2), 45 (T3) and 60 (T4) minutes. * = significantly different from T0

Systolic arterial pressure decrease was significant at all time point compared to T0; reduction of MAP was not significant 15 min after the infusion started, while it was at T2, T3 and T4. Diastolic arterial pressure reduction was significant at all time points. The effect of labetalol treatment on HR was not significant (*p* = 0.2454) (Fig. 2). Results are reported in Table 2.

**Table 2:** Effect of labetalol infusion on cardiovascular parameters, reported as mean (± SD)

	T0	T1	T2	T3	T4
SAP	175 (±25) *p* = 0.0019	152 (±38) *p* = 0.0041	150 (±28) *p* = 0.0079	144 (±28) *p* = 0.0009	142 (±30) *p* = 0.0002
MAP	130 (±22) *p* = 0.0001	113 (±28) *p* = 0.0519	108 (±20) *p* = 0.0048	101 (±19) *p* = 0.0004	98 (±20) *p* < 0.0001
DAP	108 (±20) *p* < 0.0001	90 (±25) *p* = 0.0263	85 (±16) *p* = 0.0017	80 (±14) *p* < 0.0001	77 (±17) *p* < 0.0001
HR	95 (±35) *p* = 0.2454	88 (±20)	93 (±28)	95 (±22)	98 (±24)

The*p* value in the first column indicates the overall ANOVA *p* value, while the *p* values reported under the other columns indicate the post hoc *p* values in comparison with T0. *SAP *Systolic arterial pressure, *MAP *Mean arterial pressure, *DAP *Diastolic arterial pressure, *HR *Heart rate, *T0 *Beginning of the labetalol infusion; T1, T2, T3, T4: 15, 30, 45 and 60 min after the start of the infusion, respectively

In 6/20 dogs (30%) labetalol infusion rate was halved once, in 4 (20%) twice, in 3 (15%) three times, and 4 and 5 steps reductions were made in one case each; in the remaining cases, infusion rate was kept constant. Maximum and minimum median infusion rates were 2.0 (0.5–3.6) and 0.8 (0.1-2.0) mg/kg/h respectively.

Ten (50%) dogs received acepromazine IV before and/or during the infusion (mean dose 9.2 ± 3.7 mcg/kg), and in 5 cases the drug was administered prior to labetalol in the attempt to control ABP. Maximum MAP decrease was 27.5 (10–90) and 35 (14–55) mmHg in labetalol treated dogs who received acepromazine and those who did not, respectively. The difference was not significant (*p* = 0.735).

## Discussion

This retrospective study documents the efficacy of labetalol infusion as treatment for naturally occurring acute hypertension in dogs, during and after surgery.

Perioperative hypertension is much less common than hypotension, but its consequences can be severe and its treatment challenging, in particular when the cause is not nociception. No general consensus or guidelines exists about the definition or thresholds of perioperative hypertension in veterinary medicine, but it is generally accepted to try to maintain blood pressure within the range of autoregulation (MAP between 60 mmHg and 140 mmHg) [23].

In human medicine hypertension is associated with worse surgical outcome, increased risk of myocardial ischemia, cerebrovascular accidents, bleeding, increased duration of hospitalization, and pulmonary oedema in presence of cardiac disease [24, 25]. For these reasons, treatment is recommended when elevation of ABP is greater than 20% compared to baseline [26]. In the acute setting, the treatment goal is to rapidly decrease blood pressure by no more than 25% [27], considering also the nature of the surgery, patients’ comorbidities, and risks of treatment.

The ideal agent for treatment of hypertensive emergencies should be rapid acting, predictable, easily titrated, safe and inexpensive. Intravenous infusions of antihypertensive medications present the advantage of a rapid effect, with controlled and steady lowering of blood pressure, with rapid cessation upon discontinuation of infusion [26]. When treating hypertension, it is important to rule out causes such as nociception, light plane of anaesthesia, iatrogenic causes (drugs administration, intravascular volume overload from excessive intraoperative fluid therapy), hypercarbia, and hypoxemia [28]. Nociception was considered to be unlikely as a possible cause of hypertension in the cases considered in this study prior to starting labetalol infusion, because the increases in HR and respiratory rate (during anaesthesia fighting the ventilator) that frequently accompany the higher blood pressure were not seen, and therapeutic doses of analgesics were administered. Intraoperative monitoring (expired anaesthetic agent, clinical assessment of depth of anaesthesia, capnography, pulse oximetry) were also routinely used to rule out other possible causes of hypertension. Although subjective sensitivity cannot be ruled out, the rate of fluid therapy (3.4 ± 1.5 ml/kg/h) was provided according to one of the current guidelines [29], making fluid overload extremely unlikely.

Heart rate was only slightly and not significantly (*p* = 0.2454) increased during labetalol therapy, as expected from its reversible antagonism activity at both α- and β-adrenoceptor [30]. This is in agreement with studies in humans and experimental dogs [3, 9, 20], and it is a considerable advantage, as compensatory tachycardia can delay the antihypertensive effect and increase myocardial oxygen consumption [31].

Sixty percent of the cases enrolled in our study underwent craniotomy to remove an intracranial mass; in accordance with both human and veterinary reports, this is the most frequent perioperative scenario in which treatment of hypertension is likely required [13, 14]. Hypertension, arbitrarily defined as SAP ≥ 190 mmHg and/or DAP 100 mmHg on 2 consecutive readings [32], significantly increases morbidity after this type of procedure as it is considered to predispose to intracranial haemorrhage, although a causative effect has not been fully demonstrated [15]. An acute increase in blood pressure may also damage the blood brain barrier and increase cerebrovascular permeability, predisposing to cerebral oedema [33, 34]. In an animal model, labetalol was effective in decreasing acute intracranial haemorrhage after ischemic stroke without significant systemic hypotension [35]. The effect of labetalol on cerebral blood flow is insignificant and autoregulation is preserved; the α-blocking action may prevent the sympathetic vasoconstriction of larger cerebral arteries that can compromise blood flow during a fall in blood pressure [36, 37]. Vasoconstriction of small arterioles represents a protective mechanism against overperfusion during hypertension, and vasodilation without concomitant reduction in blood pressure can contribute to worsen brain injury [38].

Hypertension after craniotomy was experienced mainly in the early postoperative period in this study; this is a complication frequently reported in humans after craniotomy [12]. It often begins 10–20 min after surgery and may last up to 4 h and, despite the cause not being well understood, it is thought to be related to increased sympathetic tone and vasoactive substances release [39]. Labetalol has been demonstrated to be effective in reducing post-craniotomy emergence hypertension, causing effective reduction in blood pressure without affecting cerebral blood flow autoregulation [40, 41]. Its action is quick after bolus administration, with onset of action of 10–20 s and peak effect after 5 min [42]. Since intravenous labetalol does not produce a significant reduction in cerebral blood flow and it is easily titrated to effect, it may be the anti-hypertensive treatment of choice in patients who also have cerebrovascular disease, considering also the fact that it does not directly affect intracranial pressure [21, 40].

In the cases examined, ABP reduction was gradual, smooth and controlled, with no undesired brisk falls and no transient hypotension, in agreement with results in humans after craniotomy, where labetalol had a predictable positive effect on haemodynamic, the stability of which is paramount in situations where cerebral flow autoregulation may be impaired [41].

Although the number of dogs was small, partly because of the low incidence of pheochromocytoma in dogs, labetalol was also effective in controlling acute hypertension during adrenalectomy in 4/5 cases, while in another one the effect was not deemed satisfactory. Surgical resection of pheochromocytomas carries a high risk of cardiovascular instability, with marked acute increases in ABP and HR due to secretion of catecholamines during induction of anaesthesia and tumour manipulation [43]. The aims of perioperative management are to prevent an acute hypertensive crisis in the operating room and then to minimize catecholamine-induced haemodynamic changes during anaesthesia and surgery. Calcium channel blockers, adrenergic receptors antagonist and magnesium sulfate are commonly used to prevent rapid changes of ABP [44]. Case reports describe the satisfactory use of labetalol before, during and after surgery in humans, with rapid and dose-related fall in blood pressure without reflex tachycardia and without significant reduction in heart rate [45, 46].

One of the dogs with pheochromocytoma presented a marked increase in SAP, MAP and DAP at the first measurement after labetalol infusion started (T1), then the parameters gradually decreased over time; in other two an initial decrease was noticed, then the pressure increased (T2) before decreasing again. A paradoxical hypertensive effect of labetalol has been reported in patients with pheochromocytoma. It has been suggested that the weak α-blockade may trigger a paradoxical increase of vascular resistance following β-blockade [47, 48]. For this reason, despite many successful reports of its use in this setting, the use of labetalol as a sole agent may not be the best option in treating intraoperative hypertension associated with pheochromocytoma in humans [49]. It remains to be established whether administration of long acting α-adrenoceptor blockers before surgery, commonly used to prepare dogs to pheochromocytoma removal, may reduce the risk of this event.

Acepromazine was administered in 10 (50%) patients at variable dose between 5 and 16 mcg/kg; in 5 dogs it was administered as first line treatment to control hypertension, in 4 dogs during labetalol infusion, and 1 dog received it in premedication. Interestingly this drug did not appear to have a significant effect on ABP control strategy. Acepromazine decreases blood pressure blocking vascular α-adrenergic receptors, with consequent vasodilation and decrease in systemic vascular resistance, making it unpopular for the management of unstable patients or those at increased anaesthetic risk [50]. Acepromazine in premedication impacts the vasopressor response of dogs to isoflurane, making them more likely to become hypotensive during anaesthesia, and it may interfere with treatments aimed at restoring normotension [51]. Despite this important side effects, it was never effective in controlling hypertension in our cases. The block of α_1_ receptor alone is probably not potent enough to control the effect of catecholamine release in these cases, or a higher dose may be needed to achieve the desired effect.

The main limitation of this study is its retrospective nature. Intra and postoperative pharmacological managements were not standardized, although type and dose of anaesthetic and vasoactive agents were similar between cases, and this may have influenced the response of the patients to labetalol. Labetalol dose was not standardized; therefore, it is not possible to characterise a dose-effect relationship, yet we have provided a dosing range that may be helpful when treating hypertension. The blood pressure value at which labetalol was started was also decided by the attending anaesthetist and is possible that response to treatment may also depend on severity of hypertension; higher values may take longer to improve, and the 60 min timeframe considered can be insufficient for a satisfactory drug effect. Patients in this study received a number of medications potentially affecting ABP, such as acepromazine, dexmedetomidine, steroids and mannitol, and it is possible that different results may have been obtained with the use of labetalol alone. On the other hand, the fact that labetalol effect was consistent and predictable across such a variety of conditions and concurrent medications, both in awake and anaesthetized dogs is remarkable and highly indicative of the titrability and efficacy of this drug in dogs. A prospective study is however warranted to further confirm our preliminary results.

## Conclusions

Labetalol was effective in decreasing ABP in dogs experiencing acute intra or postoperative hypertension. Furthermore, prospective studies are needed to better elucidate the potential role of labetalol in the treatment of hypertension.

## Abbreviations

ABP: Arterial blood pressure
SAP: Systolic arterial pressure
MAP: Mean arterial pressure
DAP: Diastolic arterial pressure
HR: Heart rate

## Availability of data and materials

The datasets generated and/or analysed during the current study are not publicly available due but are available from the corresponding author upon request.
